# *In Vivo* Analysis of the Neurovascular Niche in the Developing *Xenopus* Brain

**DOI:** 10.1523/ENEURO.0030-17.2017

**Published:** 2017-07-31

**Authors:** Melissa Lau, Jianli Li, Hollis T. Cline

**Affiliations:** 1 Graduate Program in Neurosciences, University of California, San Diego, CA 92093; 2 The Dorris Neuroscience Center, The Scripps Research Institute, La Jolla, CA 92037

**Keywords:** endfeet, neural progenitor cell, neurogenesis, neurovascular niche, optic tectum, Xenopus

## Abstract

The neurovascular niche is a specialized microenvironment formed by the interactions between neural progenitor cells (NPCs) and the vasculature. While it is thought to regulate adult neurogenesis by signaling through vascular-derived soluble cues or contacted-mediated cues, less is known about the neurovascular niche during development. In *Xenopus laevis* tadpole brain, NPCs line the ventricle and extend radial processes tipped with endfeet to the vascularized pial surface. Using *in vivo* labeling and time-lapse imaging in tadpoles, we find that intracardial injection of fluorescent tracers rapidly labels Sox2/3-expressing NPCs and that vascular-circulating molecules are endocytosed by NPC endfeet. Confocal imaging indicates that about half of the endfeet appear to appose the vasculature, and time-lapse analysis of NPC proliferation and endfeet-vascular interactions suggest that proliferative activity does not correlate with stable vascular apposition. Together, these findings characterize the neurovascular niche in the developing brain and suggest that, while signaling to NPCs may occur through vascular-derived soluble cues, stable contact between NPC endfeet and the vasculature is not required for developmental neurogenesis.

## Significance Statement

Proper development of the nervous system requires strict regulation of neural progenitor cells (NPCs), the cells that divide and generate neurons. NPCs can be influenced by extracellular cues, including potential signaling cues from the vasculature. Here, we characterize interactions between NPCs and the vasculature during brain development, and investigate potential mechanisms for vascular-mediated regulation of NPC neurogenesis. This provides insight into the diversity of regulatory mechanisms that regulate neurogenesis and ensure proper formation of the brain.

## Introduction

The finding that neural progenitor cells (NPCs) cluster around blood vessels, first observed in adult hippocampus, led to the hypothesis that vasculature-NPC association generates a unique microenvironment called the neurovascular niche (Palmer et al., 2000; Filippov et al., 2003; Seri et al., 2004). The neurovascular niche is comprised of close physical associations between NPCs and the vasculature, and in some regions of vertebrate brain, is thought to support adult neurogenesis by signaling through both contact-mediated and vascular-derived factors (Goldman and Chen, 2011). Less is known about the role of the neurovascular niche during developmental neurogenesis. In the developing brain, radial glia are the NPCs that generate post-mitotic neurons (Malatesta et al., 2000; Noctor et al., 2001; Bestman et al., 2012). The characteristic morphology of NPCs includes a soma positioned along the ventricle, and a radial process terminating in an elaborated endfoot at the highly vascularized pial surface (Bentivoglio and Mazzarello, 1999). This association of NPC endfeet with the pial surface, along with studies suggesting that the meninges are a source for pro-proliferative, pro-survival cues (Barakat et al., 1981; Gensburger et al., 1986; Radakovits et al., 2009), suggests that interactions between NPCs and the pia regulate their proliferative activity. Recent studies in the developing rodent brain identified NPCs closely associated with the vasculature, implicating NPC-vascular interactions in the regulation of neurogenesis (Tan et al., 2016) and angiogenesis (Ma et al., 2013). To date, two types of mechanisms have been proposed for how vascular signals might regulate NPC proliferation: contact-dependent cues expressed on vascular endothelial cells and soluble cues released from the vasculature. Whether the developmental neurovascular niche operates through one or both of these signaling modes is not clear.

Direct contact between NPCs and blood vessels has been observed across multiple neurogenic regions, suggesting that contact-dependent cues signal from the vasculature to NPCs. For instance, adult neurogenesis occurs in the subventricular zone (SVZ) of the forebrain (Alvarez-Buylla and Garcia-Verdugo, 2002), where NPC somata and endfeet form close associations with the vasculature (Palmer et al., 2000; Filippov et al., 2003; Seri et al., 2004; Mirzadeh et al., 2008; Shen et al., 2008; Tavazoie et al., 2008; Kokovay et al., 2010; Ottone et al., 2014; Gebara et al., 2016; Moss et al., 2016). Direct cell-cell contact between NPCs and vascular endothelial cells in the adult SVZ maintains NPC quiescence through contact-dependent signaling (Ottone et al., 2014). By contrast, in the developing mouse ganglionic eminence, NPCs associate with periventricular blood vessels, and disruption of this vascular apposition *in vivo* impairs NPC proliferation (Tan et al., 2016), suggesting that contact-dependent cues promote neurogenesis in developing cortical neurogenic regions. Interestingly, this study suggests that comparable periventricular vascular-NPC interactions do not occur in dorsal cortex, suggesting region-specific differences in neurovascular control of proliferation. It remains unclear whether contact-dependent signaling pathways in the neurovascular niche regulate proliferation across developmental neurogenic regions.

Soluble cues from the vasculature have been implicated *in vitro* to promote proliferation of both adult and embryonic NPCs (Shen et al., 2004; Arai and Lo, 2009; Plane et al., 2010). These studies suggest that the vasculature may regulate neurogenesis by signaling to NPCs by diffusible, vascular-derived factors. The neurovascular niche in the developing brain may be uniquely positioned to receive regulatory signals from the vasculature, given that the blood-brain barrier (BBB) is still forming throughout periods of developmental neurogenesis (Johanson, 1980; Kniesel et al., 1996; Liebner et al., 2008; Ben-Zvi et al., 2014; Seo et al., 2014). No *in vivo* evidence from the developmental neurovascular niche exists yet to support this model.

We were interested in whether neurovascular interactions affect neurogenesis by contact-mediated mechanisms and/or vascular-derived cues. We addressed this in *Xenopus laevis* tadpoles, which allow *in vivo* labeling and time-lapse imaging of both the vasculature and NPCs. NPCs in developing *Xenopus* tectum exhibit similar characteristics to those of other vertebrates, with somata lining the ventricle and radial processes elaborating endfeet at the pial surface (Bestman et al., 2012). Blood vessels form a stereotyped meshwork on the pial surface of the optic tectum (Rovainen and Kakarala, 1989), suggesting that association of NPC endfeet with the pial vasculature forms a neurovascular niche in the developing brain. Here, we demonstrate that NPC endfeet associate with the vasculature on the pial surface of the brain. Simultaneous *in vivo* time-lapse imaging of NPC lineages and NPC endfeet-vasculature interactions suggest that proliferative activity does not correlate with stable vascular apposition. NPC endfeet endocytose vascular-circulating molecules and NPCs accumulate fluorescent dextrans following intracardial injection, suggesting that vascular-derived cues may regulate developmental neurogenesis.

## Materials and Methods

### Animal use and care

Albino *X. laevis* tadpoles were generated by in-house breeding, while transgenic *X. laevis* tadpoles were shipped from the National *Xenopus* Resource at the Marine Biological Laboratory in Woods Hole, MA. We used the following transgenic strains: *Flk1:GFP* (RRID:NXR_0.0018) and *NBT:GFP* (RRID:NXR_0.0035) in a wildtype pigmented background. Tadpoles of both sexes were reared in 0.1× Steinberg’s solution in a 22°C incubator with a 12/12 h light/dark cycle. Transgenic tadpoles were raised as above, with the addition of 0.001% phenylthiourea to inhibit pigmentation (Hu et al., 2005). Tadpoles of both sexes were used for all experiments, staged according to Nieuwkoop and Faber (Nieuwkoop and Faber, 1956) and anesthetized before all procedures via bath application of 0.02% tricaine methanesulfonate (MS-222). All procedures were done in accordance with the regulations and approval of The Scripps Research Institute’s Institutional Animal Care and Use Committee.

### Image acquisition and processing

Anesthetized tadpoles were placed in a coverslipped Sylgard chamber for *in vivo* imaging. The majority of the *in vivo* imaging experiments were done with a PerkinElmer Ultraview Vox Spinning Disk Confocal microscope, using a 25× Nikon water-immersion lens (1.1 N.A). We used excitation lasers that were 488, 561, and 633 nm, with emission filters of 500-530, 580-650, and 660-750 nm. Images were acquired using Volocity 3D Image Analysis Software (PerkinElmer) and processed (brightness/contrast adjusted) using ImageJ. The *in vivo* pHrodo images were collected with a Nikon C2 confocal microscope, using a 20× PlanApo air objective (0.8 N.A.). We used excitation lasers that were 408 nm (diode), 488 nm (Argon ion), 543 nm (diode), and 640 nm (diode), with emission filters of 430-470, 499-529, 522-588, and 640 nm long pass. Images were acquired using NIS-Elements Advanced Research software and processed (brightness/contrast adjusted) using ImageJ. Fixed tissue for immunohistochemistry was either imaged with the Nikon C2, described above, or an Olympus FluoView500 confocal microscope, using 20× (0.8 N.A.) and 60× (1.4 N.A.) oil immersion lenses. The FluoView500 was used with excitation lasers of 488 nm (Argon ion laser), 543 nm (HeNe laser), and 633 nm (HeNe laser), along with emission filters of 505-525, 560-600, and 600 nm long pass. Images were processed with ImageJ (brightness/contrast adjustments).

### Whole-brain electroporation

Fluorescent protein reporter constructs were electroporated into the tecta of anesthetized late stage 46 tadpoles, using whole-brain electroporation . The plasmid constructs were injected (1 mg/ml) into the midbrain ventricle and 35-V pulses were applied across the midbrain with platinum electrodes flanking the midbrain. One pulse in each direction produced sparse labeling, while up to four pulses in each direction generated more widespread labeling. Following electroporation, tadpoles were returned to 0.1× Steinberg’s solution. Images through the dorsoventral extent of the tectum were collected at daily intervals from anesthetized stage 47 tadpoles starting at 24 h postelectroporation (hpe) and continued through 72 hpe, during which the tadpoles were at stage 47. The pCAG::GFP construct contains a CAG promoter, which is a strong synthetic promoter composed of a cytomegalovirus (CMV) early enhancer element, the chicken β-actin promoter and first exon and intron, and the splice acceptor of the rabbit β-globin gene (Niwa et al., 1991). The CAG promoter drives expression of GFP. The pCMV::RFP-nls reporter construct uses the CMV promoter to drive expression of turboRFP (RFP) tagged with a nuclear localization sequence (nls) to restrict the fluorescent protein to the nucleus. The pSox2bd::GFP and pSox2bd::RFP-nls reporter constructs contain Sox2/Oct3/4 enhancer elements and a minimal FGF promoter. Endogenous Sox2 or Oct-3/4 must bind to the regulatory sequence to drive expression of fluorescent protein, ensuring that only Sox2^+^Oct3/4^+^ NPCs express fluorescent protein.

### Intracardial dextran injection

Intracardial dextran injection was used for acute labeling of the vasculature in live tadpoles, as well as in assays for uptake of vascular-circulating molecules. For acute labeling of the vasculature, anesthetized tadpoles were arranged (ventral side up) under a dissecting microscope. Approximately 200-500 nl of solution containing 10,000 MW Alexa Fluor 647 dextrans (ThermoFisher Scientific, #D22914) at 25 mg/ml was pressure injected via glass micropipette into the heart. The entire volume of dextran solution is injected in a series of 5- to 10-nl pulses spaced over a minute. Tadpoles were imaged immediately after intracardial injection. For tadpoles imaged at multiple time points (with an interval of 24 h), intracardial dextran injection were performed daily. For dextran uptake experiments, 70,000 MW fluorescein dextrans (ThermoFisher Scientific, #D1822) were used (Hoffmann et al., 2011). After 5 h of recovery in 0.1× Steinberg’s solution, tadpoles were fixed and subsequently used for immunohistochemistry. For visualization of endocytic vesicles, injections were performed as described, except with 10,000 MW pHrodo red dextrans (ThermoFisher Scientific, #P10361). During the imaging interval, tadpoles anesthetized again and injected intracardially with 10,000 MW Alexa Fluor 647 dextrans (ThermoFisher Scientific, #D22914) to label the vasculature.

### DiI labeling

Late stage 46 *Flk1:GFP* transgenic tadpoles were injected in the midbrain ventricle with Vybrant DiI Cell-Labeling solution (ThermoFisher Scientific, #V2285). The tadpoles were immediately fixed in 4% paraformaldehyde in PBS, and left overnight at 4°C. Brains were dissected and whole mounted on coverslips (in 6 M urea in 50% glycerol) for confocal microscopy.

### Immunohistochemistry

Following fixation, brains were dissected and processed as whole mount or embedded in a chicken albumin gelatin mix (20% chicken albumin, 1.5% gelatin, 1% gluteraldehyde), and sectioned horizontally at 40 μm by vibratome. The sections were then quenched for 20 min at room temperature in 1% sodium borohydride in 0.5% Triton-X 100 in PBS (PBST). Following three 10-min washes in PBST, the sections were processed for immunohistochemistry in the same manner as whole mounts, as follows. Whole brains or sections were blocked in 1% normal donkey serum and 4% bovine serum albumin in PBST for 2 h at room temperature. After incubation with primary antibody (antibody-specific details to follow), samples were then washed three times in PBST, then labeled with secondary antibody diluted in block at 1:200 for 4-6 h at room temperature. After three PBST washes, samples were then mounted in 6 M urea in 50% glycerol. For colabeling with DAPI, DAPI (5 mg/ml) was added to the mounting media at 1:3000. For immunofluorescence labeling against Vimentin, samples were processed as above, except for the following: tadpoles were fixed overnight at −20°C in methanol. Following rehydration in graded methanol-PBS washes, brains were dissected out and blocked as above. Brains were incubated in primary antibodies for 3 d at 4°C, using a Vimentin antibody (Developmental Studies Hybridoma Bank, #14h7, RRID:AB_528507) at a dilution of 1:50 in block. For immunofluorescence labeling against Sox2/3, samples were processed as above, except for the following: tadpoles were fixed in 4% PFA in PBS, overnight at 4°C. Tadpoles were prepared for both whole mount and 40-μm vibratome sections. Samples were incubated in primary antibody: Sox2/3 antibody (Cell Signaling, #2748, RRID:AB_823640) at 1:200 in block, for 3 d at 4°C. For immunofluorescence labeling against HuC/D, samples were processed as above, except for the following. Samples were fixed in 1% PFA in PBS, overnight at 4°C. Whole brains were incubated in primary antibody: HuC/D antibody (Invitrogen, #A-21271, RRID:AB_221448) at 1:250 in block, for 2 h at room temperature, then 3 d at 4°C. For labeling with IsolectinB4, samples were processed as above, except for the following. Tadpoles were fixed in 4% PFA in PBS, overnight at 4°C; 40-μm vibratome sections were incubated overnight at 4°C in FITC-conjugated isolectin B4 (Sigma, #L2895; resuspended to 1 mg/ml) at 1:200 in blocking solution.

### Immunohistochemistry quantifications

Sox2/3 immunoreactivity was assessed in the dextran-labeled cells in the proliferative zone. Dextran-labeled cells were counted in two to three optical sections collected at 10-μm intervals, using the dextran-labeled anterior commissure as an anatomic landmark to identify the dorsal-most tectum. HuC/D immunoreactivity was assessed in the dextran-labeled cells enriched near the neuropil in ventral tectum. Immediately ventral of the anterior commissure, cells were counted in three to four optical sections per animal at 10-μm intervals. Dcx immunoreactivity was assessed in the dextran-labeled cells enriched near the neuropil in ventral tectum. Immediately ventral of the anterior commissure, cells were counted in four to five optical sections per animal at 10-μm intervals. For all immunolabeled populations, dextran-labeled cells were first annotated and counted in a single channel without the immunolabel visible. Dextran-labeled cells were then subsequently classified as positive or negative for the corresponding immunolabel.

### Calculating endfoot density

Vascular and nonvascular ROIs were manually traced for each tectum using maximum projections of each optical stack. For the vascular ROI, the outline of the second-most rostral blood vessel (of those that run medial-lateral) was traced manually. For the nonvascular ROIs, the outline of the dorsal surfaces immediately rostral and caudal to the blood vessel selected as the vascular ROI was traced manually. Endfeet within or partially within the drawn ROIs were counted toward the calculation of endfoot density and endfoot number was normalized to the dorsally projected surface area selected in the vascular and nonvascular ROIs for each tectum. We propose that selection of vascular and nonvascular ROIs in maximum projections provides a fairly accurate estimate of the dorsal surface area, because the brain samples are compressed by coverslips. Examination of the fluorescent signal in orthogonal optical sections provides confidence that there is minimal curvature to the samples. Likewise, examination of the *Flk1:GFP* vascular label via orthogonal optical sections provides confidence that there is minimal curvature to the blood vessels, likely due to fixation and compression of the samples.

### Calculating distances between endfoot centroids and vessel edges

Distances between endfoot centroids and vessel edges were automatically computed in 3D using Volocity 3D Image Analysis Software (PerkinElmer). Automated vessel tracing was computed on the dextran channel based on fluorescence intensity, and verified by manual inspection. Endfoot tracing was computed on the GFP channel based on fluorescence intensity, and manually inspected for each endfoot to ensure inclusion of all endfoot features, including any visible filopodia. Further, each endfoot computation was done within a manually drawn ROI that excluded the cell’s radial process from the calculations. For each endfoot computation, an endfoot centroid was defined, and the distance between the endfoot centroid and the closest vessel edge was calculated in 3D. The longest axis was also measured for each endfoot, which was then halved to provide the endfoot radius.

### Double nuclei assay

Late stage 46 tadpoles were coelectroporated with pCAG::GFP and pCMV::RFP-nls, or pSox2bd::GFP and pSox2bd::RFP-nls. At 24 hpe, each tadpole was injected intracardially with fluorescent dextran, and immediately imaged with spinning disk confocal microscopy. GFP^+^ NPC endfeet were scored for apposition to the vasculature, by checking for overlapping fluorescent signals in orthogonal cross-sections. Each NPC was classified as containing single or double nuclei.

### Clonal lineage analysis

Late stage 46 tadpoles were anesthetized and electroporated with pCAG::GFP and pCMV::RFP-nls. Immediately before each imaging time point at 24, 48, and 72 hpe, tadpoles received intracardial dextran injections and were imaged with spinning disk confocal microscopy. GFP^+^ NPC endfeet were scored for apposition to the vasculature. Nominal data (i.e., apposed vs unapposed) were collected instead of continuous data (i.e., distance from the vasculature) to score NPC interactions with the vasculature over multiple days. NPCs that maintained apposition with the vasculature over 3 d were identified as stably apposed, while NPCs that maintained lack of apposition with the vasculature were identified as stably unapposed, and all else were grouped as unstable. To summarize daily continuous measurements into a discretized ordinal variable (e.g., stably within 0-10 μm of the vasculature, stably within 10-20 μm of the vasculature) would introduce assumptions based on the arbitrarily defined thresholds. Here, we use the nominal data of apposed versus unapposed to directly examine the effect of apposition (and thus contact-mediated signaling) on neurogenic behavior. To characterize neurogenic behavior, GFP^+^ cells were also tracked and identified daily as NPCs or neurons, based on morphologic criteria. NPCs have triangular cell bodies with long, straight radial processes that terminate in elaborated endfeet at the pial surface. Neurons have rounded or pear-shaped cell bodies with long, winding axons, and branched dendritic arbors.

### Membrane-targeted horseradish peroxidase (mHRP) labeling and electron microscopy

Tadpoles were electroporated with a construct encoding mHRP, which is driven by a CMV promoter and modified with a transmembrane domain to target expression to the membrane (Li et al., 2010). The tadpoles with only NPCs labeled were then prepared for electron microscopy.

### Statistical analysis

All experiments were analyzed with the statistical tests listed in Table 1. Fisher’s exact test was used in cases with nominal variables (2 × 2 table) with small sample sizes (*n* < 1000), as recommended for improved accuracy (McDonald, 2014). Power analyses were conducted using G*Power 3.1 (Faul et al., 2009).

**Table 1.** Statistical table

	Data structure	Type of test	Power
a	Normally distributed	*t* test	0.090
b	Categorical	χ^2^ test	1.000
c	Categorical	Fisher’s exact test	0.059
d	Categorical	χ^2^ test	0.085
e	Categorical	*Post hoc* χ^2^ test with Bonferroni-corrected α	0.053
f	Categorical	*Post hoc* χ^2^ test with Bonferroni-corrected α	0.148
g	Categorical	χ^2^ test	0.097
h	Categorical	χ^2^ test	0.999
i	Categorical	*Post hoc* χ^2^ test with Bonferroni-corrected α	0.999
j	Categorical	*Post hoc* χ^2^ test with Bonferroni-corrected α	0.737
k	Categorical	*Post hoc* χ^2^ test with Bonferroni-corrected α	0.989
l	Categorical	Fisher’s exact test	0.745
m	Categorical	Fisher’s exact test	0.476
n	Categorical	Fisher’s exact test	0.059

## Results

### The neurovascular niche in developing optic tectum

**Figure 1.**
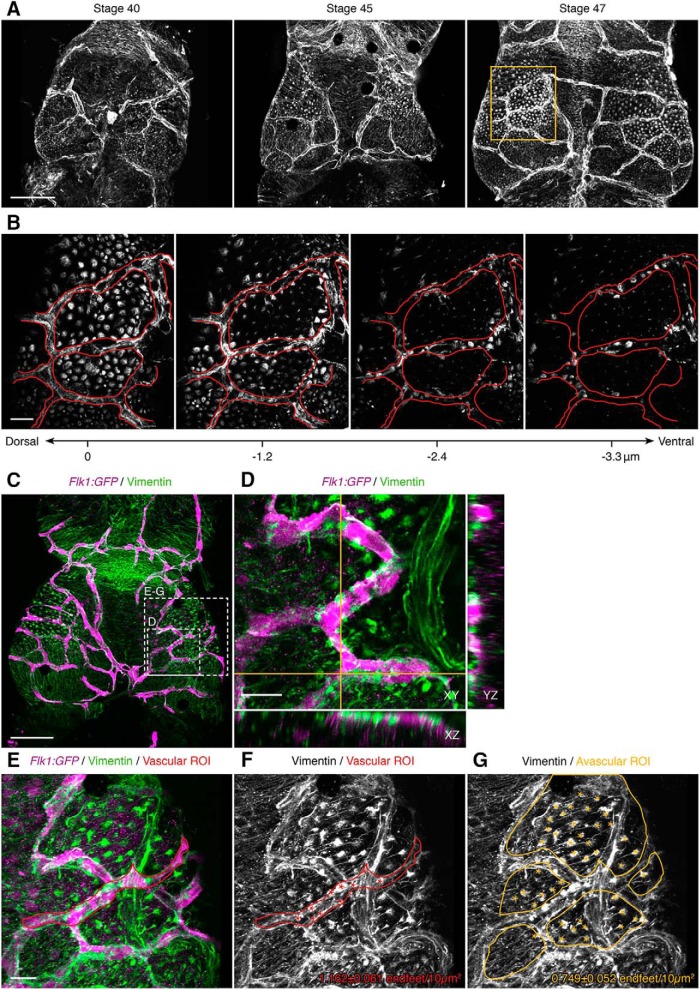
The distribution of NPC endfeet and blood vessels across the pial surface changes during development. ***A***, Vimentin-labeled blood vessels and NPC endfeet in stage 40, 45, and 47 tadpole optic tectum. Images are maximum projections of the first 50 μm from the dorsal surface of the tectum. Scale bar: 100 μm. ***B***, Higher magnification of the box in ***A***. Serial optical sections (advancing ventrally from left to right, at the indicated depths) through stage 47 tectum with Vimentin-labeled blood vessels and NPC endfeet. The vasculature is present in the two dorsal sections (left panels). The outline of the vasculature (red) is overlaid across all sections to show the distribution of Vimentin-labeled endfeet along the ventral aspect of the blood vessels (two rightmost panels). Scale bar: 20 µm. ***C***, Maximum projection image of Vimentin-labeled NPCs (green) and vasculature in a stage 47 *Flk1:GFP* (magenta) transgenic *X. laevis* tadpole. Scale bar: 100 μm. ***D***, Magnification of the labeled box in ***C*** in single orthogonal sections, showing the association between blood vessels (magenta) and NPC endfeet (green) across cross-sectional planes in XY, YZ, and XZ. Scale bar: 20 μm. ***E***-***G***, Magnification of the labeled box in ***C***, as a maximum projection, showing a representative tectum for the calculation of endfoot density. Scale bar: 20 μm. ***E***, The vascular ROI (red) was designated using the *Flk1:GFP* channel (magenta) to manually trace a blood vessel. ***F***, Overlay of the vascular ROI (red) on the Vimentin channel (white), with red asterisks indicating endfeet counts within the ROI. ***G***, Overlay of the avascular ROIs (yellow) on the Vimentin channel (white), with yellow asterisks indicating endfeet counts within the ROI.

To characterize the neurovascular niche in tadpole optic tectum we first determined the distribution of NPC endfeet and their interactions with the pial vasculature over a period of rapid neurogenesis in the optic tectum. We immunolabeled brains of stage 40-47 tadpoles for Vimentin, an intermediate filament protein enriched in NPC endfeet and processes (Tremblay et al., 2009), as well as vascular endothelial cells (Blose and Meltzer, 1981; Pixley and de Vellis, 1984). Simultaneous Vimentin immunolabeling of both NPC endfeet and blood vessels shows that the optic tectum becomes increasingly vascularized over early development, with a concomitant increase in the number and size of Vimentin-immunolabeled NPC endfeet at the dorsal surface of the tectum (Fig. 1*A*). By stage 47, endfeet tile across much of the dorsal tectal surface. In addition, serial optical sections through the dorsal tectum demonstrate that NPC endfeet are closely associated with the circumference of pial blood vessels (Fig. 1*B*).

To further examine interactions between NPC endfeet and the vasculature, we conducted Vimentin immunolabeling in tecta of transgenic *Flk1:GFP* tadpoles, in which GFP is expressed in vascular endothelial cells (Fig. 1*C*). Vimentin-immunolabeled endfeet are in close apposition to blood vessels, through all orthogonal planes of cross-section (Fig. 1*D*). The density of Vimentin-labeled endfeet is significantly higher in vascular (Fig. 1*E*,*F*), compared to neighboring avascular (Fig. 1*G*), areas of the dorsal tectal surface: vascular endfoot density, 1.16 ± 0.06 endfeet/10 µm^2^, *n* = 8; avascular endfoot density, 0.75 ± 0.05 endfeet/10 µm^2^, *n* = 8; Student’s *t* test, *p* = 0.0001_a_.

**Figure 2.**
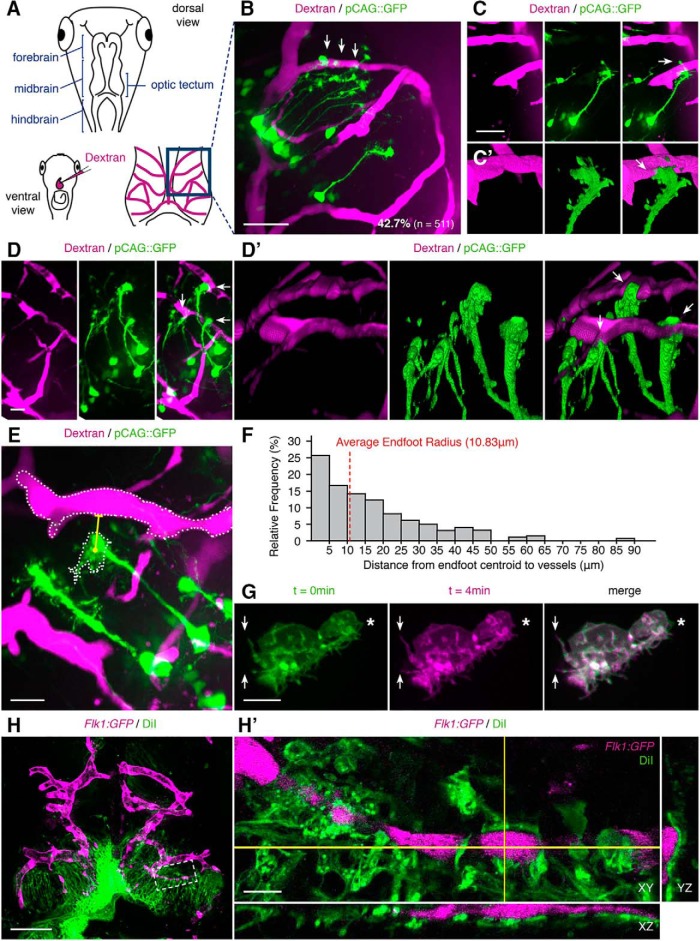
NPC endfeet appose the vasculature in the developing tectum. ***A***, Schematics of the *X. laevis* experimental system. Brain regions are indicated in the sketch of the dorsal view of the tadpole head (top). The heart and intracardial dextran injection are shown in a sketch of the ventral view (lower left) and labeled vasculature in a drawing of the dorsal tectum (lower right). ***B***, A representative image (right) of dextran-labeled blood vessels (magenta) and NPCs (green) labeled by electroporation of pCAG::GFP. 42.7% of labeled NPCs (*n* = 511) have endfeet (arrows) that are apposed to the vasculature. Scale bar: 50 μm. ***C***, ***C’***, ***D***, ***D’***, Examples of pCAG::GFP-labeled NPCs (green) whose endfeet (arrows) are apposed to the vasculature (magenta). ***C’***, ***D’***, 3D rotations of regions with NPC endfeet highlighted by arrows in ***C***, ***D***, respectively, showing vascular apposition. Scale bars: 50 and 20 μm. ***E***, Projection of a confocal stack through tectum with pCAG::GFP-labeled NPCs (green) and dextran-labeled vasculature (magenta). Automated vessel and endfoot tracing (white) on a representative cell, with the shortest distance between the endfoot centroid and vessel edge (yellow), computed in 3D. Scale bar: 20 μm. ***F***, Histogram of the distances, computed in 3D, between endfoot centroids and the closest vessel edge (*n* = 200 NPCs). ***G***, Representative *in vivo* time lapse images of a pSox2bd::mGFP-labeled NPC endfoot, collected at two time points: 0 min (green) and 4 min (magenta). The overlay in the right panel highlights rapid extensions (arrows) and retractions (asterisk) of the NPC endfoot’s filopodia. Scale bar: 10 μm. ***H***, DiI-labeled NPCs (green) in late stage 46 *Flk1:GFP* (magenta) transgenic *X. laevis* tadpoles. Scale bar: 100 μm. ***H’***, Magnification of the boxed region in ***H*** showing the association between blood vessels (magenta) and NPC endfeet (green) in single orthogonal sections of cross-sectional planes in XY, YZ, and XZ. Scale bar: 10 μm.

**Figure 3.**
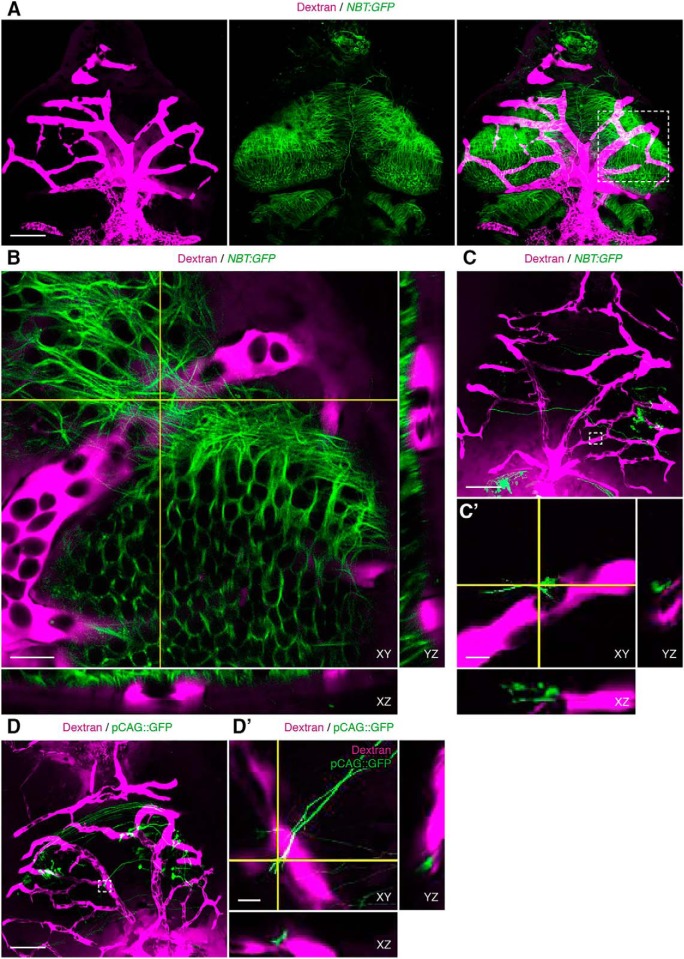
Neuronal processes and growth cones are closely associated with the vasculature in developing *Xenopus* tectum. ***A***, *In vivo* imaging of neuronal processes (green) and the vasculature (magenta) in stage 47 *NBT:GFP* transgenic *X. laevis* tadpoles. Images are maximum projections of the first 60 μm below the dorsal surface of the tectum. Scale bar: 100 μm. ***B***, Magnification of the box in ***A*** in single orthogonal sections, showing the association between blood vessels (magenta) and neuronal processes (green) across cross-sectional planes in XY, YZ, and XZ. Scale bar: 20 μm. ***C***, ***D***, Examples of GFP^+^ electroporated cells (green) and their association with dextran-labeled blood vessels (magenta). Boxes show examples of GFP^+^ neuronal growth cones that are closely apposed to the vasculature. Scale bars: 100 μm. ***C’***, ***D’***, Magnification of the boxes in ***C***, ***D***, respectively. Single orthogonal sections show association between blood vessels (magenta) and neuronal growth cones (green) across cross-sectional planes in XY, YZ, and XZ. Scale bars: 10 μm.

To examine interactions between individual NPCs and the vasculature during neurogenesis, we sparsely labeled NPCs by electroporation of a pCAG::GFP reporter plasmid, which drives strong GFP expression in NPCs. The vasculature was labeled *in vivo* by intracardial injection of fluorescent dextran (Fig. 2*A*). If an NPC endfoot had point(s) of apparent contact with the vasculature, within multiple cross-sectional views, then the NPC was classified as “apposed” to the vasculature. This analysis indicates that 42.7% of NPCs (*n* = 511 NPCs in 30 tadpoles) extend endfeet that closely appose the vasculature (Fig. 2*B–D*, arrows; Movie 1). Given that blood vessels only cover 19.4 ± 0.74% of the dorsal tectal surface (*n* = 7 tadpoles), the observed rate of NPC apposition to the vasculature is significantly higher than predicted by chance (apposed_observed_, 218 cells; unapposed_observed_, 293 cells; apposed_theoretical_, 99 cells; unapposed_theoretical_, 412 cells; χ^2^ goodness-of-fit test, *p* < 0.0001_b_). Some NPCs have branched endfeet that wrap around the sides of blood vessels (Fig. 2*C*, arrows), while other NPCs extend club-shaped endfeet that abut the vasculature (Fig. 2*D*, arrows). We also observed close apposition between neuron tectal cell axonal growth cones and the vasculature (Fig. 3). Finally, dorsal blood vessels branch and penetrate to ventral tectum (Movie 1).

Movie 1. Colabeling of vasculature, neurons, and NPCs in optic tectum. Video of a spinning disk confocal Z stack (292.4 × 292.4 μm) shows pCAG::GFP-labeled NPCs and neurons (green) with respect to the dextran-labeled vasculature (magenta) in intact *Xenopus* tectum. Each frame is a single optical section advancing at 1-µm intervals from dorsal to ventral through the confocal stack, displayed at 7 fps. At 24 h after electroporation of pCAG::GFP, tadpoles were injected intracardially with fluorescent dextran and imaged immediately. Note GFP-labeled NPC endfeet in close apposition to a blood vessel. This video is a full-frame view of the entire tectum from which Fig. 2*D*,*D’* is a cropped maximum projection. 10.1523/ENEURO.0030-17.2017.video.1

We calculated distances between pCAG::GFP-labeled NPC endfeet and the vasculature using an automated program to trace endfoot and blood vessel edges. Distances were computed in 3D between each endfoot centroid and the closest blood vessel edge (Fig. 2*E*). We found that 25.5% of endfeet centroids (*n* = 200) lie within 5 μm of a blood vessel, 16.5% of endfeet centroids lie between 5 and 10 μm of a blood vessel, and 14.0% of endfoot centroids lie between 10 and 15 μm of a blood vessel (Fig. 2*F*). Cumulatively, the majority of endfoot centroids lie within 15 μm of a blood vessel. The average radius of a pCAG::GFP-labeled endfeet (*n* = 186) is 10.83 μm. Time lapse *in vivo* imaging of NPCs expressing membrane-targeted GFP (mGFP) shows that their endfeet extend dynamic filopodia-like processes that can reach ∼10 μm (Fig. 2*G*), suggesting that these quantifications are a conservative measure of an endfoot’s spatial extent (Tremblay et al., 2009).

Widespread labeling of NPCs by ventricular injection of DiI, a lipophilic dye, further corroborates the observed apposition between endfeet and the vasculature, labeled here in transgenic *Flk1:GFP* tadpoles (Fig. 2*H*). When examined in single orthogonal sections, DiI-labeled endfeet are in close apposition to the vasculature through all planes of cross-section (Fig. 2*H’* ).

**Figure 4.**
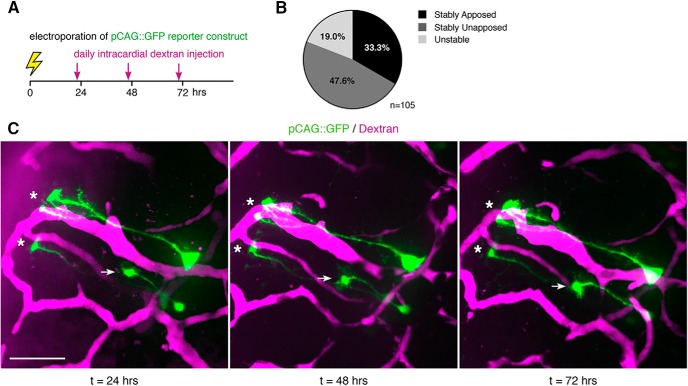
NPC endfeet maintain proximity to the vasculature. ***A***, Schematic of the experimental timeline. NPCs in late stage 46 tadpoles were electroporated with pCAG::GFP, and the vasculature was labeled by intracardial injection with fluorescent dextran just before each imaging time point at 24, 48, and 72 h after electroporation. ***B***, The percentages of NPCs (*n* = 105) that are stably apposed (33.3%), stably unapposed (47.6%), and unstable (19.0%) across 3 d of *in vivo* imaging. ***C***, A representative *in vivo* time lapse image series of pCAG::GFP-labeled NPCs (green) and dextran-labeled vasculature (magenta) collected at daily intervals over 72 h. Asterisks indicate endfeet that remain stably apposed, arrow indicates endfoot that remains stably unapposed to the vasculature across the 72-h imaging interval. Scale bar: 50 μm.

To determine the stability of NPC endfeet in close proximity to the vasculature, we imaged individual NPCs at daily intervals over 72 h in intact tadpoles. NPCs were labeled by electroporation of the pCAG::GFP reporter construct, and the vasculature was labeled by daily intracardial injections of fluorescent dextran (Fig. 4*A*). Over the 72-h period, the majority of endfeet maintained their position relative to the vasculature: 33.3% of NPCs were apposed to the vasculature at all time points, 47.6% were unapposed to the vasculature at all time points and 19.0% of NPCs changed their apposition status across the daily time points (Fig. 4*B*,*C*). Although most endfeet appear stable across the 3 d of imaging, it is likely that endfeet-vasculature interactions are dynamic over time and that endfeet retract from and subsequently reengage with the vasculature between the daily observational time points. Even so, the apparent stability of NPC-vascular interactions across daily imaging time points (Fig. 4*A–C*) suggests that NPC endfeet maintain proximity to the vasculature.

### NPC endfeet endocytose vascular-circulating molecules

**Figure 5.**
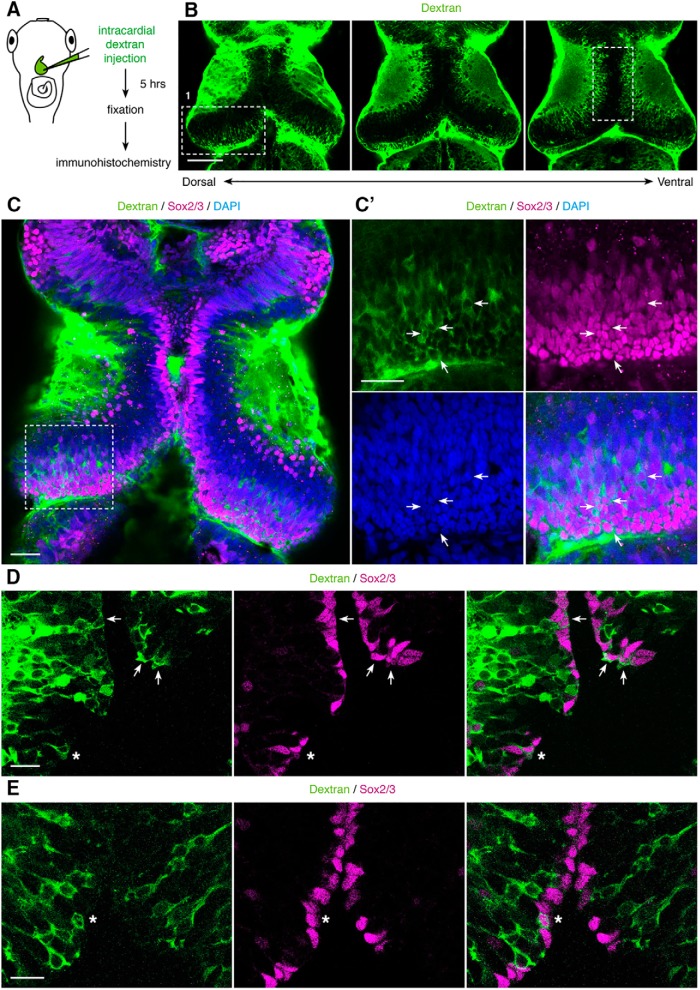
NPCs take up vascular-circulating molecules. ***A***, Schematic of the intracardial injection procedure to introduce fluorescent dextrans into the vasculature. After allowing the fluorescent dextrans to circulate for 5 h, the animals were killed and processed for immunohistochemistry. ***B***, Serial optical sections from dorsal to ventral tectum show widespread labeling of tectal cells by vascular-circulating dextrans (green). In dorsal tectum, box 1 shows dextran-labeled cells in caudolateral tectum, within the proliferative zone along the ventricle. More ventrally, box 2 shows dextran-labeled cells along the midline, also within the tectal proliferative zone. Scale bar: 100 μm. ***C***, Dextran-labeled cells (green) in the caudolateral proliferative zone are colabeled with antibodies against Sox2/3 (magenta), an NPC marker. Scale bar: 50 μm. ***C’***, Enlargement of the box from ***C***. Arrows indicate examples of Dextran^+^Sox2/3^+^ cells. Scale bar: 20 μm. ***D***, ***E***, Examples of dextran-labeled cells (green) that colabel with Sox2/3 (magenta) within the midline proliferative region. Arrows indicate Dextran^+^Sox2/3^+^ cells, and asterisks indicate Dextran^+^Sox2/3^+^ cells that are actively dividing and contain two nuclei. Scale bars: 20 μm.

**Figure 6.**
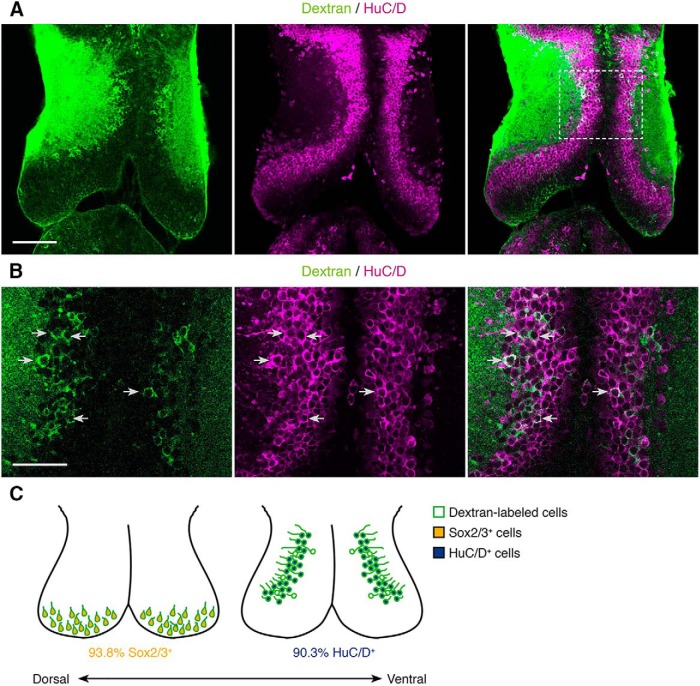
Neurons take up vascular-circulating molecules. ***A***, A single optical section through the optic tectum taken 5 h after intracardial dextran injection. Cell close to the neuropil layer that are labeled by taking up vascular-circulating dextrans (green) are colabeled with HuC/D (magenta), a neuronal marker. Scale bar: 100 μm. ***B***, Magnification of box in ***A***. Arrows indicate examples of dextran^+^ cells (green) that are also HuC/D immunoreactive (magenta). Scale bar: 50 μm. ***C***, Summary schematic of the colocalization of cell-type specific markers in dextran-labeled cells. In dorsal tectum, 93.8 ± 1.38% (*n* = 12 tadpoles) of dextran-labeled cells in the caudolateral proliferative zone are Sox2/3 immunoreactive. In ventral tectum, 90.3 ± 1.73% (*n* = 7 tadpoles) of dextran-labeled cells close to the neuropil are HuC/D immunoreactive.

**Figure 7.**
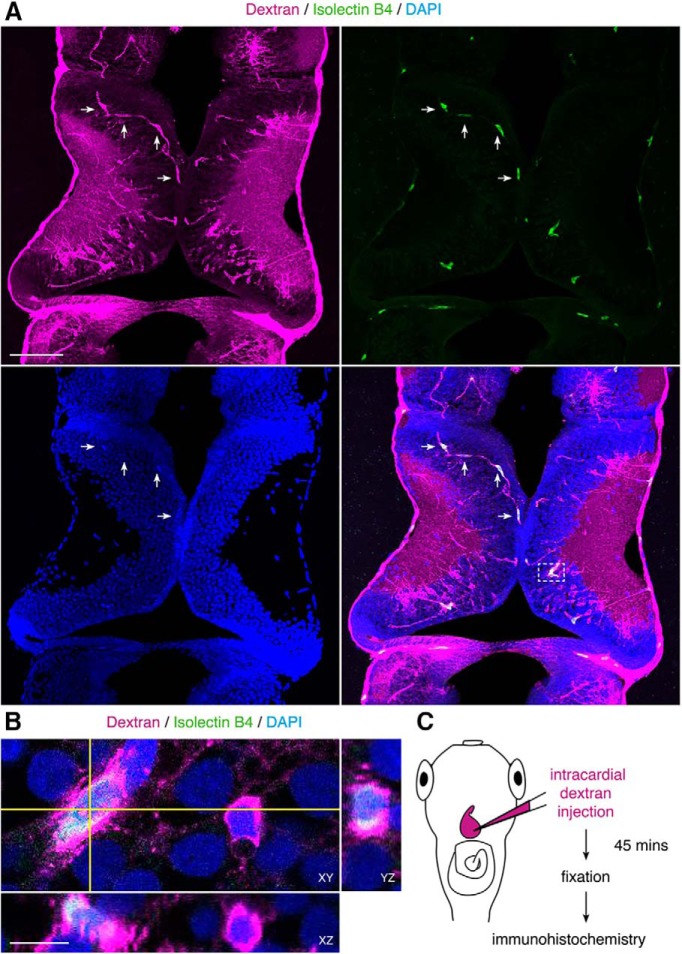
Microglia take up vascular-circulating molecules. ***A***, Projection of a 20-μm confocal stack through the optic tectum. Uptake of vascular-circulating dextrans (magenta) by tectal cells labeled with Isolectin B4 (green), a marker for microglia. Nuclei are labeled with DAPI (blue). Arrows indicate examples of Isolectin B4^+^dextran^+^ cells that track along dextran-labeled vasculature (magenta). Scale bar: 100 μm. ***B***, Magnification of box in ***A***. Single orthogonal sections show dextran (magenta) uptake in Isolectin B4^+^ cells (green), across cross-sectional planes in XY, YZ, and XZ. Scale bar: 10 μm. ***C***, Schematic of the intracardial injection procedure to introduce fluorescent dextrans into the vasculature. After allowing the fluorescent dextrans (magenta) to circulate for 45 min, the animals were killed and processed for immunohistochemistry.

*In vitro* studies suggest that vascular endothelial cells release soluble cues that promote proliferation. This has been demonstrated in coculture experiments with adult NPCs, embryonic NPCs, and oligodendrocyte precursor cells (Shen et al., 2004; Arai and Lo, 2009). To address the hypothesis that NPCs endocytose diffusible, vascular-derived signaling cues *in vivo*, we first tested whether vascular-circulating molecules are taken up by NPCs. We used *in vivo* intracardial injections to introduce fluorescent dextrans into the vasculature (Fig. 5*A*). Five hours after injection, the tectum was well labeled, with strong fluorescent signal in the tectal neuropil and cell body layers (Fig. 5*B*). Dextran uptake is concentrated in cells in the proliferative zone, which extends as a crescent from the rostromedial tectum to the caudolateral tectum. Uptake in the cells in the caudolateral proliferative zone can be seen in sections through the dorsal tectum, while dextran-labeled cells in the rostromedial tectum can be seen in more ventral sections (Fig. 5*B*, boxes 1 and 2, *C*,*D*). Immunolabeling for Sox2/3, transcription factors expressed in NPCs, demonstrates that vascular-circulating dextrans are taken up by Sox2/3^+^ NPCs in the proliferative zone lining the tectal ventricle (Fig. 5*C–E*; Movie 2). Actively dividing NPCs containing two Sox2/3^+^ nuclei are labeled with dextran (Fig. 5*D*,*E*, asterisks). Neurons, identified by the pan-neuronal marker HuC/D, and microglia, labeled with Isolectin B4, are also labeled with vascular-circulating fluorescent dextrans (Figs. 6, 7). Together, these data indicate that NPCs, neurons, and microglia take up vascular-circulating molecules.

Movie 2. Uptake of vascular-circulating molecules in the tectum. Video of a 60-μm confocal stack, progressing from dorsal to ventral tectum. Five hours after intracardial injection of fluorescent dextrans (green), tadpoles are fixed and processed for immunohistochemistry against Sox2/3 (magenta). Dextran-labeled cells in the caudolateral proliferative zone are colabeled with Sox2/3, an NPC marker. Other dextran-labeled cells, lacking Sox2/3 immunoreactivity, are primarily located near the neuropil. 10.1523/ENEURO.0030-17.2017.video.2

**Figure 8.**
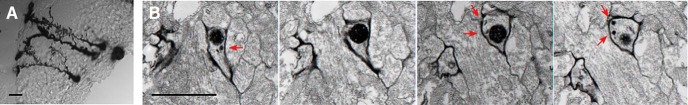
NPC endfeet are sites of active endocytosis. ***A***, Image of mHRP-labeled NPCs. Scale bar: 1 μm. ***B***, Micrographs from serial electron microscopic sections demonstrate the ultrastructure of an mHRP-labeled endfoot. Arrows indicate mHRP-labeled multivesicular organelles within the labeled endfoot, consistent with endocytosis in endfeet. Scale bar: 1 μm.

To determine if vascular-circulating molecules are taken up by endocytosis, we first tested whether endocytosis occurs at NPC endfeet. To label endocytic vesicles, we expressed mHRP in NPCs, which labels the plasma membrane and allows for visualization of endocytic vesicles at the ultrastructural level (Li et al., 2010). Here, we employed this technique to label NPCs in *Xenopus* tectum, assaying for the presence of mHRP-labeling within NPC endfeet. We find that mHRP reliably labels membranes of NPC endfeet, and that mHRP-labeled multivesicular organelles are present within endfeet (Fig. 8). These mHRP-labeled organelles are much larger than synaptic vesicles in the surrounding axon terminals (Fig. 8). The data are consistent with a model of internalization of endocytic vesicles at NPC endfeet, however mHRP also labels vesicles being trafficked toward the cell surface, for example from the Golgi to the plasma membrane. Therefore, we used an alternate labeling method to specifically identify internalized vesicles containing vascular-circulating molecules.

**Figure 9.**
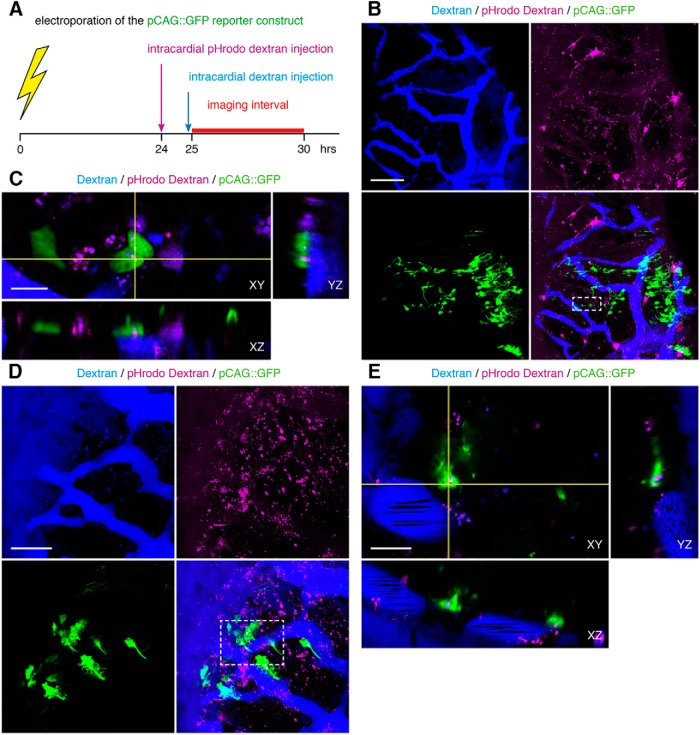
NPC endfeet endocytose vascular-circulating molecules. ***A***, Schematic of the experimental timeline. Late stage 46 tadpoles are electroporated with pCAG::GFP to label NPCs, and then intracardially injected with pHrodo dextran 24 h later to label endocytic vesicles with the vascular-derived pHrodo dextran. Within the next few hours, tadpoles are injected again with fluorescent dextran to label the vasculature, and immediately imaged. ***B***, Maximal projection image of a 40-μm confocal stack through tectum with intracardial injections of dextran (blue) and pHrodo dextran (magenta). In images collected 1.5 h after pHrodo dextran injection. pHrodo^+^ puncta identify endocytic vesicles (magenta) in pCAG::GFP-labeled NPCs (green) that appose the vasculature (blue). The merged image (lower right) shows the distribution of pHrodo^+^ puncta relative to the vasculature. Scale bar: 100 μm. ***C***, Enlargement of box in ***B***, lower right. Single orthogonal sections, showing the presence of pHrodo^+^ puncta (magenta) within NPC endfeet (green) that appose the vasculature (blue), across cross-sectional planes in XY, YZ, and XZ. Scale bars: 10 μm. ***D***, Projection of a 30-μm confocal stack through tectum with intracardial injection of dextran (blue) and pHrodo dextran (magenta). In images collected 3.5 h after pHrodo dextran injection, pHrodo^+^ puncta label endocytic vesicles (magenta) in pCAG::GFP-labeled NPCs (green) that do not appose the vasculature (blue). The merged image (lower right) shows the distribution of pHrodo^+^ puncta relative to the vasculature. Scale bar: 50 μm. ***E***, Enlargement of box in ***D***, lower right. Single orthogonal sections, showing pHrodo^+^ puncta (magenta) within NPC endfeet (green) that do not appear to appose the vasculature (blue), across cross-sectional planes in XY, YZ, and XZ. Scale bars: 10 μm.

**Figure 10.**
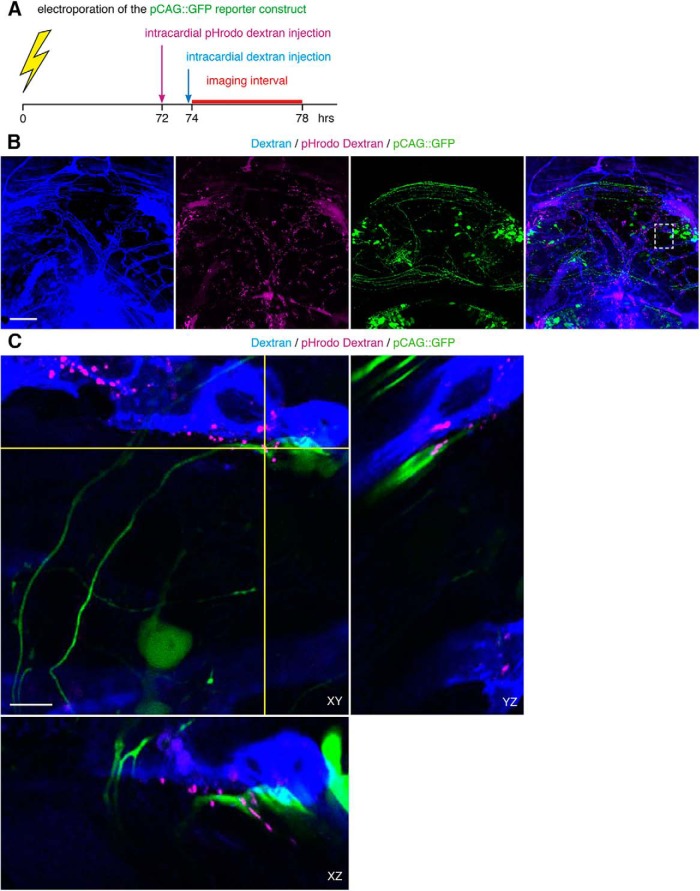
Neuronal growth cones endocytose vascular-circulating molecules. ***A***, Late stage 46 tadpoles are electroporated with pCAG::GFP to label neurons, and then intracardially injected with pHrodo dextran 72 h later to label vascular-derived endocytic vesicles. After 2 h for circulation, tadpoles are injected again with dextran to label the vasculature, and immediately imaged. ***B***, Projection of a 40-μm confocal stack through tectum with intracardial injections of dextran (blue) and pHrodo dextran (magenta). In images collected 2 h after pHrodo dextran injection, pHrodo^+^ puncta identify endocytic vesicles (magenta) in pCAG::GFP-labeled cells (green) relative to the vasculature (blue). Merged image shows relative location of enlarged neuronal growth cone in ***C***. Scale bar: 100 μm. ***C***, Enlargement of box in ***B***. Single orthogonal sections demonstrate the presence of pHrodo^+^ puncta (magenta) within a neuronal growth cone (green) that apposes the vasculature (blue), across cross-sectional planes in XY, YZ, and XZ. Scale bars: 10 μm.

To test whether vascular-circulating dextrans are endocytosed at NPC endfeet, we used intracardial injections of pHrodo dextrans to visualize endocytic vesicles *in vivo* (Fig. 9*A*). pHrodo dextrans are pH sensitive, such that they only fluoresce when internalized into acidic compartments, including endocytic vesicles. Endocytic vesicles rapidly acidify to pH 6.0-6.5 within minutes of endocytosis (Tycko et al., 1983), and eventually fuse with endosomes that reach pH 4.5-5.5 (Sorkin and Von Zastrow, 2002). pHrodo dextran fluoresces in both compartments. We detected pHrodo^+^ puncta in the tectum within hours after intracardial injection (Fig. 9*B–E*). At high magnification, pHrodo^+^ puncta are detected within NPC endfeet that closely appose the dextran-labeled vasculature, as well as endfeet that do not appear to appose the labeled vasculature, seen when viewing orthogonal planes of cross-section (Fig. 9*C*,*E*). We detect pHrodo^+^ puncta immediately adjacent to the dextran-labeled vasculature, indicating endocytic vesicles that are either within unlabeled cells that appose the vasculature, or within the vascular endothelial cells of the blood vessels (Fig. 9*B–E*); the latter suggests transcytosis as a possible mechanism for the extravasation of vascular-circulating dextrans. pHrodo^+^ puncta are also detected within neuronal growth cones (Fig. 10). Taken together, these data demonstrate that NPC endfeet and neuronal growth cones endocytose vascular-circulating molecules.

### Apposition between NPC endfeet and the vasculature does not correlate with NPC proliferation

**Figure 11.**
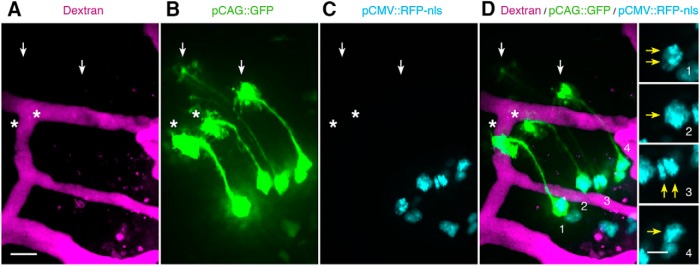
Actively dividing NPCs are not preferentially apposed to the vasculature. ***A***-***D***, Maximum projections of (***A***) the dextran-labeled vasculature (magenta), (***B***) pCAG::GFP-labeled NPCs (green) and (***C***) pCMV::RFP-nls^-^labeled nuclei (cyan), and (***D***) the merged image. Double nuclei in a single cell identifies actively dividing NPCs. Asterisks indicate NPC endfeet (green) that are apposed to the vasculature (magenta). Arrows (white) indicate NPC endfeet that are unapposed to the vasculature. The far right panel shows magnifications of cells labeled 1-4 in ***D***, with arrows (yellow) indicating the nuclei. From top to bottom, annotated cells contain double, single, double, and single nuclei (cyan). Scale bars: 20 and 10 μm.

To evaluate the possibility that contact-mediated signaling between NPC endfeet and the vasculature regulates neurogenesis in the developing optic tectum, we tested whether NPC proliferation correlates with endfoot-vascular apposition. We coexpressed cytosolic GFP and nuclear-targeted RFP_nls_ in NPCs and labeled the vasculature by intracardial injection of fluorescent dextran. We first used single-time point *in vivo* imaging to identify individual GFP^+^ NPCs with and without apposition to the vasculature and identified actively dividing NPCs by the presence of two nuclei within individual NPC somata (Fig. 11). We found that comparable proportions of NPCs were actively dividing, regardless of apposition to the vasculature (apposed: 30.7%, *n* = 153; unapposed: 32.1%, *n* = 190; *p* = 0.816_c_). Although this indicates that NPC proliferation does not correlate with apposition to the vasculature, it does not rule out the possibility that apposition may influence the type of neurogenic events that occur. For example, *in vitro* evidence suggests that vascular-derived cues stimulate embryonic NPCs to undergo symmetric divisions that expand the progenitor pool, rather than asymmetric divisions that produce neurons (Shen et al., 2004).

**Figure 12.**
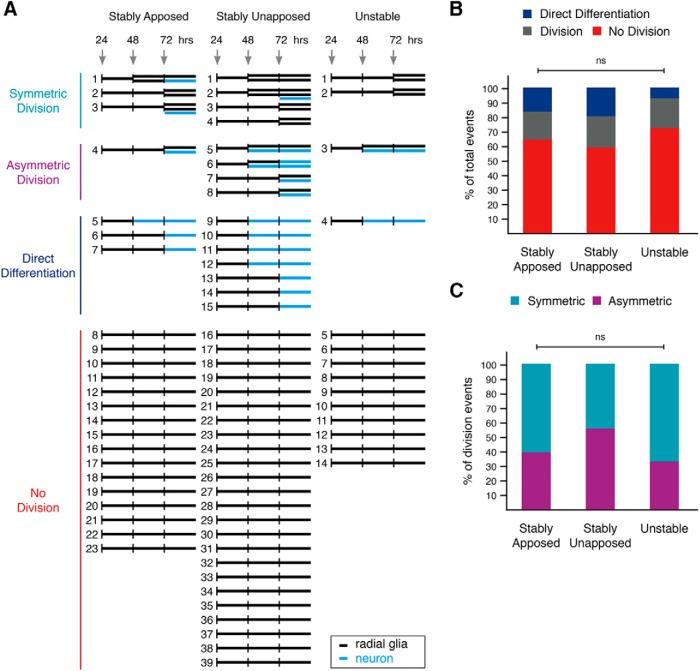
Vascular apposition does not bias NPCs toward specific neurogenic events. ***A***, Clonal lineages of individual NPCs imaged at 24, 48, and 72 h after electroporation. Each NPC is represented as a horizontal black line, which can split into two horizontal lines to represent a division event. Blue horizontal lines indicate neuronal progeny that were either generated via asymmetric division or direct differentiation of an NPC. ***B***, Proportions of neurogenic events in NPCs whose endfeet are either stably apposed (*n* = 23 cells), stably unapposed (*n* = 39 cells), or unstable (*n* = 14 cells) with respect to the vasculature. No significant (ns) differences by χ^2^ test (*p*_d_ = 0.8609). ***C***, Proportions of asymmetric and symmetric division events in dividing NPCs whose endfeet are either stably apposed (*n* = 5 cells), stably unapposed (*n* = 9 cells), or unstable (*n* = 3 cells) with respect to the vasculature. No significant (ns) differences by χ^2^ test (*p*_g_ = 0.7454).

To test whether apposition to the vasculature biases NPCs toward certain neurogenic events, we conducted *in vivo* clonal lineage analysis. 76 individual NPCs, labeled by electroporation with the pCAG::GFP reporter construct, were imaged *in vivo* at daily intervals over 72 h to track division events and identify progeny (Fig. 12*A*). NPCs were classified based on their endfoot interactions with the vasculature into the following groups: stably apposed, stably unapposed, or unstable NPCs. The majority of NPCs in all three groups remained NPCs and did not divide (stably apposed, 64.0%; stably unapposed, 58.5%; unstable, 71.4%). The neurogenic NPCs underwent symmetric divisions, asymmetric divisions, and direct differentiation events in which NPCs directly converted into neurons (Bestman et al., 2012). We found no significant difference in the proportions of neurogenic events across the three NPC groups (Fig. 12*B*, *p* = 0.861_d_). Division occurred at comparable rates across all apposition groups (stably apposed, 20.0%; stably unapposed, 22.0%; unstable, 21.4%; *p* = 0.982_e_, Bonferroni-corrected α = 0.0167), as did rates of direct differentiation (stably apposed, 16.0%; stably unapposed, 19.5%; unstable, 7.1%; *p* = 0.556_f_, Bonferroni-corrected α = 0.0167). Further, the proportions of symmetric to asymmetric divisions did not differ between NPC groups (percentages for stably apposed, stably unapposed, and unstable, respectively; symmetric: 60.0%, 44.4%, 66.7%; asymmetric: 40.0%, 55.6%, 33.3%; Fig. 12*C*, *p* = 0.745_g_). Clonal lineage analysis of pCAG::GFP-labeled NPCs demonstrates that the majority do not divide or differentiate over a 72-h imaging protocol (Fig. 12*A*,*B*), suggesting that these NPCs are slowly dividing or quiescent. While vascular apposition does not correlate with proliferative behavior in these relatively quiescent NPCs, it is possible that vascular apposition has greater effect on more proliferative NPCs.

**Figure 13.**
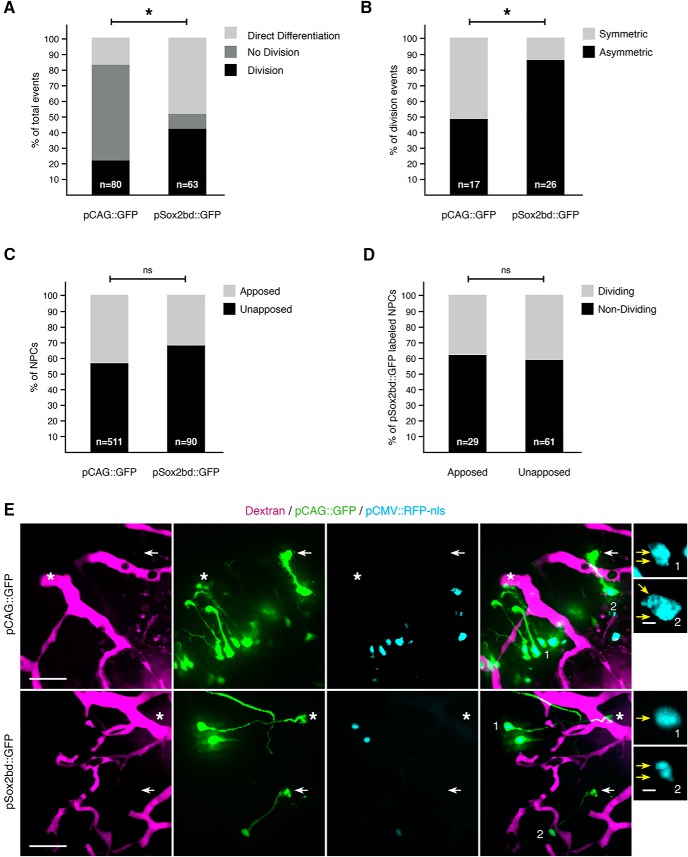
Vascular apposition does not correlate with proliferation or differentiation in a neurogenically active subpopulation of NPCs. ***A***, ***B***, Comparison of neurogenic activity between two labeled populations of NPCs. ***A***, Percentages of neurogenic events in NPCs labeled with either pCAG::GFP (*n* = 80 division events) or pSox2bd::GFP (*n* = 63 division events). Asterisk indicates a significant difference between the two NPCs populations via χ^2^ test (*p*_h_ < 0.0001). ***B***, Percentages of symmetric and asymmetric division events undergone by dividing NPCs labeled with either pCAG::GFP (*n* = 17 cells) or pSox2bd::GFP (*n* = 26 cells). Asterisk indicates a significant difference by Fisher’s exact test (*p*_l_ = 0.0162). ***C***, Percentages of NPCs that are apposed or unapposed to the vasculature, between different populations of labeled NPCs. No significant (ns) differences between pCAG::GFP (*n* = 511 cells)- and pSox2bd::GFP (*n* = 90 cells)-labeled NPCs, by Fisher’s exact test (*p*_m_ = 0.0809). ***D***, Proportions of dividing and nondividing pSox2bd::GFP-labeled NPCs. Percentages for dividing and nondividing NPCs, respectively: apposed: 37.9%, 62.1%; unapposed: 41.0%, 59.0%. No significant (ns) difference between populations of NPCs whose endfeet are either apposed (*n* = 29 cells) or unapposed (*n* = 61 cells) to the vasculature, by Fisher’s exact test (*p*_n_ = 0.8217). ***E***, Electroporation of pCAG::GFP and pCMV::RFP-nls (top row) or pSox2bd::GFP and pSox2bd::RFP-nls (bottom row) to label NPCs (green) and their nuclei (cyan), to evaluate their association with dextran-labeled blood vessels (magenta). Asterisks indicate NPC endfeet that are apposed to the vasculature, while arrows indicate NPC endfeet that are unapposed to the vasculature. The far right panel shows magnifications of cells numbered in the preceding panels. From top to bottom, the annotated NPCs contain double, double, single, and double nuclei. Scale bars: 50 and 5 μm.

To test whether a more neurogenically active population of NPCs is more sensitive to effects of vascular apposition, we imaged NPCs labeled by expression of the pSox2bd::GFP construct, containing a regulatory domain that requires binding of endogenous Sox2 and Oct3/4, transcription factors involved in self-renewal and pluripotency (Bestman et al., 2012). pSox2bd::GFP-labeled NPCs are more neurogenically active than pCAG::GFP-labeled NPCs (Fig. 13*A*, *p* < 0.0001_h_). We compared our dataset of pCAG::GFP-labeled NPCs (Fig. 12) to a previously published dataset (Bestman et al., 2012) of pSox2bd::GFP-labeled NPCs. Significantly fewer pSox2bd::GFP-labeled NPCs are quiescent over a 72-h imaging protocol (pSox2bd::GFP, 9.5%; pCAG::GFP: 61.5%; *p* < 0.0001_i_, Bonferroni-corrected α = 0.0125). A significantly greater proportion of pSox2bd::GFP-labeled NPCs divide (pSox2bd::GFP, 41.3%; pCAG::GFP, 21.8%; *p* = 0.0095_j_, Bonferroni-corrected α = 0.0125) and undergo direct differentiation (pSox2bd::GFP, 49.2%; pCAG::GFP: 16.7%%; *p* < 0.0001_k_, Bonferroni-corrected α = 0.0125). Among those division events, a greater proportion of pSox2bd::GFP-labeled NPCs undergo asymmetric divisions than the pCAG::GFP-labeled NPCs (pSox2bd::GFP: 84.6%; pCAG::GFP: 47.1%; Fig. 13*B*, *p* = 0.016_l_).

Despite these differences in proliferative behavior, we do not detect a significant difference in the rate of vascular apposition between pCAG::GFP and pSox2bd::GFP-labeled NPC endfeet (Fig. 13*C*): 42.7% of pCAG::GFP-labeled NPC endfeet and 32.2% of pSox2bd::GFP-labeled NPC endfeet appose the vasculature (*p* = 0.081_m_). Moreover, in a direct test of whether actively dividing pSox2bd::GFP-labeled NPC endfeet preferentially appose the vasculature, we found that a comparable proportion of dividing pSox2bd::GFP-labeled NPCs were apposed and unapposed to the vasculature (Fig. 13*D*, *p* = 0.822_n_). Taken together, these data indicate that stable apposition to the vasculature does not correlate with proliferation or differentiation.

## Discussion

Here, we describe the developmental neurovascular niche using *in vivo* labeling and imaging techniques to examine the relation between NPCs and the vasculature in *Xenopus* tadpole optic tectum. We demonstrate that the majority of Sox2/3-immunolabeled NPCs accumulate vascular-circulating molecules and that vascular-circulating reporters are endocytosed by NPC endfeet. Although a substantial fraction of NPCs appose the vasculature at their endfeet, neurogenic behavior does not appear to correlate with stable NPC endfeet-vascular apposition. These observations support a model in which developmental neurogenesis does not require contact between NPC endfeet and the vasculature, but may be regulated by secreted signaling cues.

### The neurovascular niche in the developing and adult CNS

We characterize the neurovascular niche during developmental neurogenesis in *Xenopus* tectum, where *in vivo* confocal imaging indicates that NPC endfeet appose the pial vasculature and maintain proximity with blood vessels over several days. This finding suggests that the neurovascular niche, described by interactions between NPCs and the vasculature, is a conserved feature of neurogenic regions across species and across brain regions. For example, NPCs appose the vasculature in the mammalian adult SVZ (Capela and Temple, 2002; Mirzadeh et al., 2008; Shen et al., 2008; Tavazoie et al., 2008; Ottone et al., 2014), adult hippocampus (Palmer et al., 2000; Filippov et al., 2003; Seri et al., 2004; Moss et al., 2016), and embryonic cortex (Ma et al., 2013; Tan et al., 2016), as well as in zebrafish (Ulrich et al., 2011) and avian species (Goldman and Chen, 2011).

Despite the conserved presence of the neurovascular niche, the architecture of the niche differs widely between brain regions and across development. NPCs in the developing *Xenopus* brain associate with the pial vasculature predominantly at their endfeet and the tectal vasculature has not yet formed a periventricular meshwork around NPC somata, similar to the staggered developmental time course of the elaboration of pial and periventricular vasculature in the mammalian ganglionic eminence (Tan et al., 2016). NPCs in the mouse ganglionic eminence, which generate cortical inhibitory interneurons, associate with periventricular blood vessels, and disruption of this vascular apposition *in vivo* impairs NPC proliferation (Tan et al., 2016). Interestingly, this study suggests that comparable periventricular vascular-NPC interactions do not occur in dorsal cortex, suggesting region-specific differences in the neurovascular control of proliferation (Tan et al., 2016). Further, pial and cerebral blood vessels have different tight junction and molecular properties (Allt and Lawrenson, 1997; Cassella et al., 1997; Lawrenson et al., 1999; Jeong et al., 2008), suggesting that neurovascular interactions may also differ with pial versus periventricular vascular association.

By contrast, the architecture of the neurovascular niche in the adult SVZ and the morphologies of the cells in the niche are significantly different from those in the developing tadpole and mouse brains (Shen et al., 2008; Ma et al., 2013; Silva-Vargas et al., 2013; Tan et al., 2016). The adult SVZ is a complex structure composed of a polarized vascular network with which the somata and processes of multiple neural cell types closely associate as they form chains of cells that migrate, proliferate, and eventually populate the olfactory bulb. The neurovascular niche in adult hippocampal dentate gyrus, the SGZ, also has a distinct architecture, in which radial glial NPC somata and endfeet contact the periventricular and pial vasculature, respectively. These differences in architecture may underlie the unique characteristics of each neurovascular niche. For example, pial and periventricular blood vessels have been shown to differ in astrocytic coverage (Allt and Lawrenson, 1997), BBB permeability (Jeong et al., 2008), paracellular tight junction separation (Cassella et al., 1997), and protein expression (Lawrenson et al., 1999), which are all properties that may affect neurovascular signaling.

Although interfering with NPC endfoot-vascular interaction may cause NPCs in embryonic mammalian cortex to die (Radakovits et al., 2009), our observations that half of NPCs in tadpole tectum are not associated with the vasculature, and that vascular apposition does not affect NPC proliferation, suggests that interactions with the vasculature are not required for NPC viability or proliferation in *Xenopus* brain. Despite repeated observations that NPCs maintain close association with the vasculature in different neurogenic regions in both developing and adult systems, the function of these interactions and the cellular basis of the interactions may differ in different neurogenic regions. For instance, laminin-integrin contact-mediated interactions between NPCs and the vascular enthothelial cells in the adult SVZ negatively regulate NPC proliferation *in vivo* (Shen et al., 2008), whereas comparable contact-mediated interactions promote proliferation in the developing ganglionic eminence (Tan et al., 2016), suggesting developmental and region-specific signaling pathways between NPCs and the vasculature regulate neurogenesis (Ottone et al., 2014).

### Soluble vascular-derived signaling cues in the neurovascular niche

We provide *in vivo* evidence that vascular-circulating molecules are endocytosed by NPC endfeet. Our data indicate that soluble signaling cues from the vasculature are endocytosed by NPC endfeet, supporting a model in which secreted cues from the vasculature affect developmental neurogenesis. *In vitro* studies demonstrate that vascular endothelial cells release soluble cues that promote self-renewal of embryonic NPCs (Shen et al., 2004). Similar coculture experiments show that soluble cues from vascular endothelial cells also increase survival and proliferation of oligodendrocyte precursor cells (Arai and Lo, 2009). Our *in vivo* evidence of endocytosis of vascular-circulating molecules by NPC endfeet provides a potential mechanism for vascular-mediated regulation of developmental neurogenesis.

Vascular-derived molecules can likely penetrate mammalian embryonic cortex and affect neurogenesis, since the BBB is still forming during developmental neurogenesis. Studies disagree on when the BBB becomes fully functional, and this point is further complicated by observations that BBB development varies between brain regions. For example, earliest estimates indicate that the BBB in cortex is fully functional by embryonic day E15.5 (Ben-Zvi et al., 2014), while other studies suggest that BBB development is a protracted process continuing throughout developmental neurogenesis, such that adult function of the BBB may occur as late as P21 in the cerebellum (Johanson, 1980). The formation and maintenance of the BBB requires interactions between vascular endothelial cells and the neighboring cells within the local microenvironment, including pericytes, astrocytes, and neurons that collectively form the neurovascular unit (Chow and Gu, 2015). For example, sonic hedgehog (Shh) is secreted by astrocytes, and required for BBB properties including the presence of tight junctions (Alvarez et al., 2011). Likewise, oligodendrocyte precursor cells secrete TGF-β1, which increases BBB integrity by upregulating tight junction proteins in endothelial cells (Seo et al., 2014). Given that both astrocytes (Molofsky and Deneen, 2015) and oligodendrocyte precursor cells (Kessaris et al., 2006) do not appear in the cortex until relatively late stages of development, the cortical BBB is unlikely to be fully formed at these stages. This provides an opportunity for vascular-circulating cues to exit the vasculature and signal to NPCs during developmental neurogenesis. Our data suggest that the neurovascular niche, formed by association between NPC endfeet and the pial vasculature, does not regulate developmental neurogenesis. While we demonstrate that NPC endfeet endocytose vascular-circulating molecules, consistent with a potential role for vascular-secreted cues, it is unclear whether endogenous vascular-derived signaling cues regulate neurogenesis. To identify potential signaling candidates, future investigations can gain insight from signaling pathways described for adult neurogenesis.

During adult neurogenesis, strong evidence indicates that soluble cues regulate proliferation, and some of the signaling pathways have been identified. First, the neurovascular niche in adult SVZ hosts a modified BBB (Tavazoie et al., 2008). Tavazoie and colleagues demonstrate that contact between NPCs and blood vessels occurs at sites lacking astrocyte, NPC endfoot, or pericyte coverage. Further, they find that vascular-circulating molecules can penetrate into the SVZ, despite their exclusion elsewhere in the brain. Second, *in vitro* experiments demonstrate that vascular endothelial cells promote self-renewal in NPCs from adult SVZ, and that, ischemic-like conditions can change the profile of vascular secreted cues such that differentiation is promoted over proliferation (Plane et al., 2010). Similarly, tumor vasculature secretes pro-proliferative cues to cancerous NPCs in the adult brain (Calabrese et al., 2007; Gilbertson and Rich, 2007). Third, secreted cues from the vasculature are also implicated in regulation of NPCs across a variety of functions. SDF-1, a chemokine protein secreted by vascular endothelial cells, guides migration of adult NPCs toward the vasculature (Kokovay et al., 2010), and also alters NPC morphology in embryonic spinal cord, following endocytic signaling via CXCR4 (Mithal et al., 2013).

Finally, some of the most convincing data that vascular cues can regulate proliferation come from observations in adult hippocampus. The finding that exercise not only increases neurogenesis and angiogenesis in the adult hippocampus (van Praag et al., 1999; van Praag et al., 2005; Ekstrand et al., 2008; Clark et al., 2009; Van der Borght et al., 2009), but also enhances NPC contact with the vasculature (Gebara et al., 2016), led to the hypothesis that vascular-circulating factors promote proliferation. Several growth factors are elevated in blood after exercise, even in humans (Schwarz et al., 1996; Schobersberger et al., 2000), and were found to increase neurogenesis when introduced into the vasculature or brain (Tao et al., 1996; Tao et al., 1997; Wagner et al., 1999; Aberg et al., 2000). Indeed, two such circulating growth factors, VEGF and IGF-1, are required for the exercise-induced increase in hippocampal neurogenesis (Fabel et al., 2003; Trejo et al., 2001). Both are potential candidate signaling cues to test in the context of developmental neurogenesis. Future work could assay the effects of knockdown or introduction of candidate signals into the bloodstream. Additional candidates include BDNF signaling, which also regulates proliferation in the adult hippocampus (Lee et al., 2002; Sairanen et al., 2005; Scharfman et al., 2005; Li et al., 2008; Chan et al., 2008; Choi et al., 2009), and has been implicated *in vitro* to promote proliferation during developmental neurogenesis (Islam et al., 2009; Chen et al., 2013). Further, BDNF is also expressed and secreted by vascular endothelial cells in the brain, acting both as a migration cue for neuroblasts during adult neurogenesis (Snapyan et al., 2009) and as a neuroprotective signal during developmental neurogenesis (Guo et al., 2008).

Studies of heterochronic parabiosis provide additional support for the idea that vascular-derived factors affect neurogenesis. With heterochronic parabiosis, the vasculature of a young animal is joined to that of an older animal, so they share the same circulatory system. Surprisingly, circulating cues from the blood of young animals rejuvenate tissues in older animals, by increasing proliferation in many cell types (Conboy et al., 2005; Ruckh et al., 2012), including NPCs in adult hippocampus (Villeda et al., 2011; Villeda et al., 2014), and SVZ (Katsimpardi et al., 2014). Like the exercise paradigm mentioned above, the increased neurogenesis induced by blood borne factors from young animals is accompanied with concomitant improvements in behavioral and cognitive function, as well as significant vascular remodeling (Villeda et al., 2011; Katsimpardi et al., 2014; Villeda et al., 2014). Together, heterochronic parabiosis experiments demonstrate that circulating factors in the blood affect many aspects of cell proliferation across a range of tissues, including adult neurogenic regions.

### Contact-mediated signaling in the neurovascular niche

Studies in the adult SVZ indicate that vascular association with neural cells in the neurovascular niche, for instance mediated by laminin/integrin interactions (Shen et al., 2008), or interaction between Eph receptor tyrosine kinases and Ephrin-B ligands (Conover et al., 2000; Ottone et al., 2014), negatively regulate NPC proliferation *in vivo*. Whether contact-mediated NPC-vascular interactions regulate neurogenesis in the developmental neurovascular niche is still not clear. In our study, analysis of NPCs *in vivo* showed that vascular apposition with NPC endfeet did not correlate with NPC proliferative activity or differentiation in *Xenopus* tectum. This is consistent with studies demonstrating that detachment of NPCs from the pial surface, and thus necessarily, the vasculature, does not disrupt proliferation during embryonic neurogenesis (Halfter et al., 2002; Hartfuss et al., 2003; Haubst et al., 2006). In contrast to our study and the study in adult SVZ, interfering with integrin function in NPCs in the embryonic ganglionic eminence decreases association with the vasculature and decreases proliferation *in vivo* (Tan et al., 2016). Other studies in mouse embryonic cortex indicate that NPC survival depends on their attachment to the meninges, which contain blood vessels (Radakovits et al., 2009). Several possibilities could explain these different results. One possibility is that NPCs in developing neurogenic regions may be heterogeneous in their proliferative capacities, as described for NPCs in adult neurogenic regions (Gebara et al., 2016). For instance, we and others have reported different NPC morphologies, response properties, and molecular signatures (Tremblay et al., 2009; Bestman et al., 2012; Bestman et al., 2015; Whittington et al., 2015). A related possibility is that there may be distinct developmental time windows during which vascular-mediated regulation of neurogenesis is critical. Studies in a variety of species demonstrate stronger vascular effects earlier in development. For example, in chicks, cultured NPCs proliferate more in the presence of meningeal cells, including blood vessels, but only if the NPCs were derived from 6 d embryos or younger (Barakat et al., 1981). Studies in mouse embryonic cortex show a stronger vascular effect on NPC survival at earlier time points (E11-E13) when the majority of NPCs undergo symmetric divisions, and the vascular survival effect is abolished by E15, when NPC divisions become primarily asymmetric (Radakovits et al., 2009). Contact-mediated NPC-vascular interactions may affect NPC proliferation at earlier stages of tadpole development, or in the caudolateral tectal proliferative zone where symmetric divisions are prominent (Herrgen and Akerman, 2016).

While direct contact between NSCs and blood vessels has been confirmed at the ultrastructural level in adult hippocampus (Filippov et al., 2003; Moss et al., 2016) and adult SVZ (Tavazoie et al., 2008), previous studies (Shen et al., 2008; Ma et al., 2013; Tan et al., 2016), and our work here, rely on confocal microscopy to demonstrate close apposition between NPCs and the vasculature during development. Demonstration of direct contact requires the resolution of electron microscopy. Furthermore, NPC endfeet are studded with filopodia which extend and retract over the time course of minutes (Tremblay et al., 2009), suggesting that direct contact between NPC endfeet and the vasculature may be transient and challenging to demonstrate. The endocytosis of vascular-derived soluble fluorescent reporters, as reported above, may be a signature of transient contact between NPC endfeet and the vascular endothelial cells.

It is possible that both contact-dependent and soluble cues act simultaneously on the same or different NPCs populations within a neurovascular niche (Silva-Vargas et al., 2013), as also suggested by a morpholino-based screen of candidate neurogenic regulators (Bestman et al., 2015), such that only a subset of vascularly apposed NPCs responds to specific contact-mediated signaling. Multiple signaling systems could have opposing downstream effects targeted to different cell types. For instance, Ottone and colleagues suggest that in adult SVZ, contact-mediated vascular signaling maintains quiescence of type B NSCs, while secreted vascular cues promote proliferation in transit-amplifying type C cells that are close to (but lack contact with) blood vessels (Ottone et al., 2014). Indeed, many neurogenic regions, including developing *Xenopus* tectum, contain a mix of NPCs with and without apposition to the vasculature (Shen et al., 2008; Tavazoie et al., 2008). Further work examining NPC endfoot-vascular interactions will be important to elucidate cellular mechanisms regulating developmental neurogenesis *in vivo*.

In conclusion, we describe the neurovascular niche during developmental neurogenesis in the *Xenopus* optic tectum, in which NPC endfeet associate with the pial vasculature. We provide *in vivo* evidence that NPC endfeet endocytose vascular-circulating molecules and that stable vascular apposition does not correlate with NPC proliferation. Our studies highlight properties of the developmental neurovascular niche that are distinct from those described for the adult neurovascular niche, suggesting a diversity of roles for neurovascular interactions.

## Synthesis

Reviewing Editor: Cheryl Sisk, Michigan State University Decisions are customarily a result of the Reviewing Editor and the peer reviewers coming together and discussing their recommendations until a consensus is reached. When revisions are invited, a fact-based synthesis statement explaining their decision and outlining what is needed to prepare a revision will be listed below.

Reviewers were in agreement that the manuscript reports interesting data and that the imaging was exceptionally beautiful. However, both expressed concern that the data are primarily descriptive and anecdotal, and without additional manipulations to disrupt the molecule signaling and/or the NPC vascular apposition, the conclusions are not convincing. The primary argument seems to be that because there is no contact-mediated signal from vasculature to NPC, and because NPCs can endocytose stuff that started out in the bloodstream, there must therefore be a freely diffusible signal from the vasculature to the NPC that regulates proliferation. But no direct evidence is provided for regulation of proliferation. Discussion of a neurovascular niche in Xenopus was viewed as speculative is in light of the negative results, and is too definitively stated as an actual thing that exists with certainty.

Both reviewers indicated that if you could identify and disrupt the putative diffusible signal, then this would be an outstanding paper. If it is not possible to do this within two months (i.e., for the manuscript to be reviewed as a revision and not a new manuscript), then it is necessary to temper the conclusions in a way that does not go beyond the descriptive nature of the work. In addition, a revised manuscript should consider and respond to the specific comments below.

1. The images are lovely, but in places rather chaotic and do not seem to make the point of specificity of interactions very clearly. For example observations about the density of NPC endfeet at vasculature are not clearly demonstrated in the example images provided. Better examples should be shown, or at least the authors should use arrowheads and zoomed images to take the reader through the observation explicitly-- as is, it is far from obvious despite the very significant stats provided.

2. Many of the examples designed to show indirect communication between the vasculature and the NPCs actually show an interaction with all cell types in the tectum. This tells us that material in the vasculature does have the potential to extravise, contact and be taken up by the NPCs (as well as by neurons and microglia) but that does not mean that there is a meaningful signaling event resulting from this exchange of material. Indeed the negative result, indicating that proximity to blood vessels does not correlate with NPC proliferation, is equally if not more consistent with the idea that NPC proliferation is entirely independent of any vascular signal in the Xenopus optic tectum.

3. In the results section, most of the statistical analyses are run using categorical variables. It is not clear why this decision was made. There should be more explanation as to why the statistics were conducted the way they were. With data like the rate of apposition or number of neurogenic events it could be more telling to analyze the data as interval variables instead of categorically. With the imaging done there should be a rich dataset of information, so there should a solid justification for the statistical analyses used. Additionally, Fisher's Exact Test is used when you have an expected frequency of less than 5, however looking at their n's it looks like for all tests there should be more than 5. If these are the correct tests to use the authors need to be more transparent in their explanations. There are also many times in the results section that percentages are presented but no analysis to substantiate the claim that they are comparable or not. For instance on lines 429-430, 7.1% very well could be different from 16% and 19.5%.

4. It is not really adequate to rely on data from mammalian brain or other models as proof that in Xenopus there MUST therefore also be similar vascular-derived signaling. This must be demonstrated directly, for example by showing that a VEGF-knockdown, resulting in disrupted vascularization of the optic tectum, does indeed impact tectal NPC proliferation in some way that is correlated with the degree of devascularization. Alternatively if the nature of the putative vasculature-derived diffusible signal can be identified, knocking that down, or adding it to the bloodstream, would be quite convincing. In its current form the manuscript provides some interesting insights into potential communication between the vasculature and brain cells in the developing tectum in Xenopus but it does not really show that this is meaningful. As it stands the only firm conclusion from this study is that the BBB is very leaky in young tadpoles and can release stuff that gets taken up by all cells in the brain (especially neurons and microglia).

5. Something that could strengthen this paper would be to know what the vascular-derived molecules endocytosed by the NPCs are. If this is beyond the scope of the project it would be beneficial to specifically discuss what the authors think the molecules could be and future directions to investigate them.

6. Trafficking experiments with mHRP are equally consistent with the vesicular mHRP being contained in a compartment trafficking TOWARD the cell surface (e.g. Golgi) rather than AWAY from the surface. In other words couldn't this be surface delivery rather than internalization. The phRodo experiments help mitigate this but this point could be made more explicitly.

7. It would be valuable to measure relative area/volume occupied by vasculature in order to state whether interactions with NPC endfeet are random, more or less frequent than predicted by chance.

8: Beginning on line 321 pg.10 percentages are listed for the percentage of endfeet centroid located within three different distances from blood vessels. These percentages add up to more than 100%, does that mean the endfeet located 5μm and 10μm are included in the percentage of endfeet within 15μm? If so that seems misleading.

9. On line 430 pg. 13 there is a claim that 7.1% is comparable to 16% and 19.5%, however there is no analysis to back up that claim.

10. Line 328: Tremblay et al, 2009 is cited 3 times in the same sentence. Line 404: ‘cytosolic’. Line 491: “form” should be changed to “formed”.
